# A Time-Dose Model to Quantify the Antioxidant Responses of the Oxidative Hemolysis Inhibition Assay (OxHLIA) and Its Extension to Evaluate Other Hemolytic Effectors

**DOI:** 10.1155/2014/632971

**Published:** 2014-08-27

**Authors:** M. A. Prieto, J. A. Vázquez

**Affiliations:** Group of Recycling and Valorization of Waste Materials (REVAL), Research Marine Institute (IIM-CSIC), Eduardo Cabello 6, 36208 Vigo, Spain

## Abstract

The development of a convenient mathematical application for testing the antioxidant potential of standard and novel therapeutic agents is essential for the research community to perform evaluations in a more precise form. The* in vitro* oxidative hemolysis inhibition assay, despite its relevance for* in vivo* responses, lacks a proper mathematical model to quantify the responses. In this work, a simple nonlinear time-dose tool to test the effectiveness of antioxidant compounds is presented. The model was verified with available experimental data from the bibliography. The model helps to describe accurately the antioxidant response as a function of time and dose allowing comparisons between compounds. Its advantages are a simple application, provision of parametric estimates that characterize the response, simplification of the protocol, economization of experimental effort, and facilitation of rigorous comparisons among the effects of different compounds and experimental approaches. Finally, other effectors that may obstruct or be of interest for the antioxidant determination are also modeled in similar principles. Thus, the basis of more complex multivariable models is provided. In all experimental data fitted, the calculated parameters were always statistically significant, the equations prove to be consistent, and the correlation coefficient of determination was in all cases higher than 0.98.

## 1. Introduction

Antioxidants (A) and prooxidants are chemical entities that can delay or accelerate oxidation processes. Living organisms have developed a complex network [1, 2] of enzymatic and nonenzymatic A, which are essential to counteract various harmful prooxidants or reactive species (i.e., O_2_, H_2_O_2_, ROO^•^, and OH^•^) [3, 4]. Apart from these endogenous A, exogenous ones can derive from natural sources (vitamins, flavonoids, anthocyanins, and some mineral compounds). Clinical trials and epidemiological studies have established an inverse correlation between the intake of natural exogenous A and the occurrence of oxidative stress diseases such as inflammation, cardiovascular problems, cancer, and aging-related disorders [5–7]. Thus, the analysis of natural antioxidants for disease prevention and the identification of possible prooxidant substances have become topics of growing interest [8–10].

Several* in vivo* and* in vitro* methods have been developed for determining the A properties of compounds in order to rank their affinity. In general, these assays differ in their mechanism to generate different radical species and/or target molecules and in the way end-products are measured. At present, there is no convenient assay that enables the evaluation of the antioxidant capacity (AC) for different compounds [5, 11, 12]. The current methods to test the AC still have left many open questions [13, 14]. The* in vitro* assays can only rank AC for their particular reaction system and their relevance to* in vivo* activities is uncertain. Thus, it is logical that, in the last decade, researchers have claimed more cell based approaches [5, 15–17]. Additionally, the arbitrary use of simple analytical procedures to calculate molecular properties, occasionally without a validation study, as well as a lack of statistical significance, has caused much controversy [18–23].

OxHLIA is a bioassay for evaluating the inhibition capabilities of A [24], half way between* in vivo* and* in vitro* methods. Sheep erythrocytes are subjected to hemolysis by the action of hydrophilic and lipophilic radicals in aqueous system. Hydrophilic radicals are generated from the thermal decomposition of AAPH (2,20-azobis(2-methyl-propionamidine) dihydrochloride) attacking the erythrocytes membranes. Lipophilic radicals are derived from the oxidation of the erythrocytes membranes (lipid peroxidation), a phenomena that is initiated by the action of AAPH and thermal induction. The lipophilic and hydrophilic radicals eventually cause the hemolysis of the cell. The time at which the hemolysis occurs depends on the resistance of the erythrocytes population. This hemolytic time can be retarded by antioxidants, capturing the hydrophilic and/or lipophilic radicals. The advantages of OxHLIA are that radicals and substrate targets are biologically relevant compared to other* in vitro* methods and that antioxidants are subjected to oxidants with different degrees of polarity [25].

Originally, the OxHLIA was performed in a test-tube format, and the degree of hemolysis was spectrometrically determined in the supernatant after centrifugation [24]. Those steps impeded researchers to test large numbers of samples at the same time. Since the development of the method, several studies have improved and extended the applicability of the method [26–29]. Recently, authors [30] have performed a further key modification, which allows following the degree of hemolysis via turbidity of the erythrocyte suspension without centrifugation. This improvement enhances the applicability of OxHLIA into a microplate format. The method thus performed enables evaluation of large numbers of samples of small quantity at the same time with satisfactory precision and reproducibility and in an equivalent way to the previous format procedures.

Its main weakness is the insufficient formalism due to the lack of formal model to describe the kinetic erythrocyte hemolysis, which prevents the quantification of its statistical reliability and loses a part of the relevant information that can be drawn from the experimental results. Therefore, the quantification relies on graphical, or similar methodologies, which causes low reproducibility of the results and leads to an accumulation of procedural restrictions that overstandardize the protocol [31]. Although the meticulous results can be found concerning the kinetics and the factors affecting the reproducibility of the method, the quantification of the results has been left as it was postulated originally [24].

In the current study, a nonlinear mathematical application for describing the OxHLIA responses is developed. It helps to describe accurately the response as a function of time and dose, to provide parameters that summarize the responses, and to facilitate convenient comparisons of the AC of different compounds. In addition, by analyzing the data of other authors that have studied the effects of other variables or agents over the kinetic erythrocyte population curve, more complex analysis for AC of antioxidants is suggested. Therefore, the basis for postulating more complex multivariable models is also provided. Because the available methodological studies of the OxHLIA appear to have reviewed and extended its procedure into a robust methodology, no improvements of the protocol have been performed.

## 2. Material and Methods

### 2.1. The OxHLIA Procedure

As recommended by Takebayashi et al. [32], erythrocytes need to be obtained from several adult sheep to reduce the influence of individual differences. Although the common procedure uses sheep erythrocytes, other sources such as human or bovine ones have been proved to produce similar results. Erythrocytes should be washed at least three times with PBS and resuspended in PBS at a 2.8% (v/v) suspension.

#### 2.1.1. Test-Tube Format

As described [32], sheep erythrocytes are suspended at a final concentration in which the maximum hemoglobin liberated cannot saturate the spectrophotometer measuring. The solution is prepared in 0.7% (v/v) phosphate-buffered saline (PBS, pH 7.4) with ~40 mM of AAPH in the absence (control) and presence of increasing concentrations of antioxidant sample in a test tube at 37°C in a water bath with shaking. An aliquot of this mixture was periodically withdrawn every 15 min for 3 h, and the degree of hemolysis (%) was determined from the concentration of hemoglobin in the supernatant after centrifugation by measuring the absorbance at 524 nm. The results are expressed as delayed time of hemolysis (Δ*t*) as follows:

(1) $$\begin{array}{c} \Delta t=\tau_{S}-\tau_{C}, \end{array}$$

where *τ* is the time (min) needed to reach the 50% of the erythrocyte population that is lysed. The subscripts *S* and *C* stand for the antioxidant sample and the control, respectively.

#### 2.1.2. 96-Well Plate Format

As described by Takebayashi's et al. work [30], the erythrocyte suspensions (50 *μ*L), in the absence or presence of an antioxidant sample (100 *μ*L) in PBS, were added to a flat bottom 96-well plate. Complete hemolysis was obtained by adding water to the erythrocyte suspension without sample. The plate was preincubated with a lid at 37°C; then, AAPH (50 *μ*L, 160 mM in PBS) was added to initiate the assay and incubated at 37°C in the incubator with shaking. The optical density at 660 nm was measured every 10 min. The percentage of survival erythrocyte population (*P*) was calculated as follows:

(2) $$\begin{array}{c} P=\left(\frac{n_{t}-n_{\max}}{n_{0}-n_{\max}}\right)\times 100, \end{array}$$

where *n* is the optical density measure at the start of the reaction (0) or at any *t* (min) and *n*_max⁡_ is the maximum optical density of the complete hemolysis. Then, the time to reach 50% of the survival population (*τ*) is obtained graphically for an increasing concentration of an antioxidant. Afterwards, (2) is used to normalize the *τ* values formulated by (1).

#### 2.1.3. Quantification

The kinetic part is solved in general by a graphical method. In the test-tube and microplate approaches, the Δ*t* values obtained are analyzed in liner terms as a function of the antioxidant concentration. The slope ([A]/min) of such analysis is used as the comparative value between different compounds. Although such a double approach is correct, it seems contradictory that the algebraic tools are reserved only to the second part of the procedure, which prevents the statistical validation of the results as a whole. The statistical information of the parameters found in the first graphical step is lost, and even if we compute the parametric estimations of the second step, it does not take into account the variable involved in the first step. Thus, among other problems, the two-step procedure lacks a proper statistical estimation.

### 2.2. Data for Model Verification

The bibliographical abundance about antioxidant activity in a competitive reaction, in raw and purified extracts, makes it practically superfluous to extend the experimental work, specifically devoted to validate the model proposed here. In this respect, its descriptive accuracy was verified using results from other authors (taken from the published figures by means of* GetData Graph Digitizer 2.24*), selected in such a way that they implied different methods, substrates, and time domains.  Table 1 shows a detailed description of the references used, the corresponding figures subjected to analysis, the variable or agent involved, and some relevant conditions of the assays. As it will be noted, the studies from which the data was collected are those that had been involved in developing, reviewing, or extending the methodology responses evaluated here. We hope that the reader would find them as key data, rather than a lack of experimental effort.

### 2.3. Numerical and Statistical Methods

Fitting the experimental results to the proposed equations was carried out in two phases. First, parametric estimates were obtained by minimization of the sum of quadratic differences between observed and model-predicted values, using the nonlinear least-square (quasi-Newton) method provided by the macro* Solver* in* Microsoft Excel 2003*, which allows quick testing of hypotheses and the display of their consequences. Next, the determination of the parametric confidence intervals and model consistency (Student's *t*-test and Fisher's *F*-test, respectively, in both cases with α = 0.05) was calculated using the “*SolverAid*” [33]. The “*SolverStat*” macro [34, 35] was used for detecting possible anomalies in the distribution of parametric estimates and residuals.

## 3. Results and Discussion

In general, the bioassay studied here is of a special elegance and applicability. One of the major drawbacks is the lack of formal model to describe the kinetic erythrocyte hemolysis. Therefore, the quantification of the hemolytic time *τ* (time to reach the 50% of the survival population of erythrocytes) has to be obtained graphically or similarly from the kinetic erythrocyte survival curve and thus, the results provided lack of relevant statistical information. Additionally, such a lack of mathematical expression causes low reproducibility of results, which often leads to accumulation of procedural restrictions that could overstandardize the protocol. Despite the existence of very rigorous results about the kinetics and the factors affecting the reproducibility of the method, for some reasons, the quantification of the results has been left as it was postulated originally [24].

Next, we will briefly review different mathematical methodologies from related fields of study, such as the antioxidant, dose-response, and hemolytic bioassays. Afterwards, based on such analysis, a simple illustrative bivariate (simultaneous analysis of time and effector responses) mathematical application for many different effector agents that affect the hemolytic process is presented. Then, a highly appropriated alternative model for OxHLIA is verified by using data from other authors that had investigated the capacity of several antioxidants in the OxHLIA reactions. Finally, other effectors that may obstruct or be of interest for antioxidant determination are modeled in similar principles as those defined in the OxHLIA reaction.

### 3.1. Modeling Approaches from Related Fields of Study

Besides other authors [41–43], we reject the simplistic ways of characterization of antioxidant action and we attempt to address this issue by bringing across well-established ideas from existing fields to overcome the existing problems for the quantification OxHLIA antioxidant bioassay.  Table 2 shows a short review of different mathematical expressions from related fields of study that provide the key knowledge to guide the development of appropriate solution for the OxHLIA antioxidant bioassay. At present, the reaction mechanism of the erythrocyte lysis is well understood. Authors have developed a sophisticated mechanistic model to evaluate the erythrocyte lysis [39]. The detailed mechanistic description of oxidation is complex because the heterogeneity of the cell population varies from one to the other systems making it difficult to characterize the responses and measure all the compounds involved in the reaction. Therefore, researches tend to search for empirical general models [28, 40], able to quantify and evaluate the complex phenomenon of hemolysis. In this regard, authors [38] have demonstrated the suitability of the Weibull survival distribution connecting its parameters to blood properties.

From the antioxidant field, due to the complexity of the oxidative reactions, multiple number of simple calculation procedures and different mechanistic or empiric kinetic models [41–43, 48] are routinely used to compute the inhibitory responses of different agents. Among the available nonlinear models, the power function developed by Terpinc and Abramovič [42] is only appropriate to adjust fractional-order kinetic profiles, but it fails in the description of first-order processes or sigmoidal profiles. The Logistic and Weibull equations, which have been transferred from microbiology and pharmacology dose-response fields to describe the oxidation action [41, 43], are more appropriate equations for modeling oxidation processes.

From the dose-response theory, the three-parameter sigmoidal group of functions (such as the Logistic, Weibull, Hill, Gompertz or Richards-Chapman) would be, in general, acceptable solutions to fit individually the kinetic profiles corresponding to a series of increasing level agents or variables[44–47].

In general, most of the above models can be transferred to study the erythrocyte survival analysis found in the OxHLIA method and help to compute, in a proper form, the inhibitory features over the control curve produced by any antioxidant agent. Those models would be able to produce key parameters to summarize the responses, such as the asymptote, half-life, maximum velocity, or the lag-phase, and they can be used to quantify the effect of different chemicals. However, in the following, by merging all the above solutions, we will describe in detail what we consider as suitable solution for the OxHLIA antioxidant bioassay.

### 3.2. Mathematical Modeling for the Kinetic Description of the Survival Erythrocyte Population

#### 3.2.1. Normalization

In an open system, in the presence of enough quantity of initiator (AAPH) and in the absence of an antioxidant, the peroxidation of the erythrocyte membrane would be oxidized exhaustively at sufficiently long times, causing its rupture, producing asymptotic responses that would correspond to the erythrocyte population mixed in the solution. Consequently, the spectrophotometric responses (*R*) should be carefully standardized. Therefore, before the data is analyzed, the response (*R*) must be normalized as a function of the maximum hemolysis (*R*_max⁡_) that can be achieved. For example, a simple way could be to compute the *R* in terms of survival population (*P*) in percentage as follows:

(3) $$\begin{array}{c} P=\left(\frac{R}{R_{\max}}\right)\times 100. \end{array}$$

Normalization of the raw data through multiplication adjusts the resulting sample variation (human error, excessive or defective dilution, number of red blood cells that are present, and so forth). Fitting the normalized data resulted in a significant increase in the reproducibility and accuracy of the results obtained, but no significant change in the parameters was produced.

#### 3.2.2. Kinetic Description of the Survival Erythrocyte Population (Univariate Approach)

The above previous works [28, 38, 40] reflect as the most appropriate alternative the use of sigmoidal profiles to describe the kinetic hemolysis of erythrocytes. In accordance with other authors, we have also found that the Weibull survival distribution [49] is a suitable solution for the analysis of OxHLIA survival responses. The function can be formulated in terms of the variables *t* (time) and *P* (survival erythrocyte population) in its original form as follows:

(4) $$\begin{array}{c} \frac{P}{100}=\exp\left[-{\left(\frac{t}{b}\right)}^{a}\right], \end{array}$$

where *a* and *b* are parameters of form and position, respectively. Its use, in our context, requires two modifications: (1) to multiply the second member by the maximum response *K*, to assure that the asymptote can take values different from 100% in case any antioxidant or other variable may be able to protect a partial erythrocyte population from hemolysis and (2) to reparametrize the equation in such a way that it would include explicitly the time for the substrate half-life (*τ*), which is the desirable parameter in this type of bioassay. Therefore, the time course of the survival population of erythrocytes can be postulated to occur as function of the parameters variation as in the following equation:

(5) $$\begin{array}{c} P=K\exp\left[-\ln2{\left(\frac{t}{\tau }\right)}^{\alpha }\right], \end{array}$$

where *K* is the asymptote, *τ* is the time required for 50% oxidation (substrate half-life), and *α* is shape parameter related to the maximum slope of the response. Equation (5) is very versatile: when *α* < 1, it can adjust the profiles of potential responses; when *α* = 1, a first-order kinetic is described; when *α* > 1, a variety of sigmoidal profiles are produced.

#### 3.2.3. Additional Parameters of Interest

Apart from the *τ* parameter, other interesting ones can be obtained, such as the maximum rate of hemolysis (*v*_*m*_), the rate at the *τ* value (*v*_*τ*_), and the lag-phase (*λ*). The rate values can be obtained with some algebraic modifications from (5) as presented in Table 3 (part A). Regarding the appearance of a lag-phase, since no induction period was observed in micelle model systems, this feature of the profile in the OxHLIA survival responses is attributed [50] to the action of antioxidants present in the erythrocyte membrane. The confidence intervals of *v*_*m*_, *v*_*τ*_, and *λ* could be estimated by means of the reparameterization of (5) to make such values explicit (Table 3, part A), but the result is less operative than the robust estimation of the *τ* parameter [31].

#### 3.2.4. Application to Bibliographic Antioxidant Data

**Figure 1:**
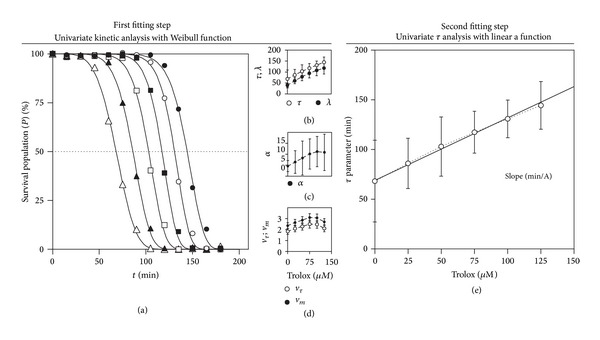
Kinetics of the haemolytic oxidation at different antioxidant concentrations and relationships among the concentration of antioxidant and the parameters that characterize its activity. (a) The kinetic series of the survival erythrocyte population fitted (lines) to the kinetic model (5) using the antioxidant trolox at various concentrations 0-(25)-125 *μ*M (dots). The results were obtained from the study of Takebayashi et al. [26] (Case A1 in Table 1) who recently made a detailed revision of the method. Parametric estimations are showed in Table 4. (b), (c), and (d) show the pattern of the parametric responses as a function of the antioxidant concentration. The parameters *τ* and *λ* show a linear behavior, while the rate is the modified final *v*_*τ*_ and *v*_*m*_ displaying a decreasing asymptotic hyperbolic relation.

Figure 1(a) shows the typical time-dose response of hemolysis curves using the antioxidant trolox at various concentrations 0-(25)-125 *μ*M (dots). The results were obtained from the study of Takebayashi et al. [26] (Case A1 in Table 1) who recently published a detailed revision of the method. The collected data was normalized by (3) and fitted (lines) to the kinetic survival function (5). Parametric estimations are shown in Table 4. Statistically significant results were found continuously (Student's *t*-test, *α* = 0.05), the equation was consistent (Fisher's *F*-test), and the goodness of fit coefficient of determination was higher than 0.98. Figures 1(b), 1(c), and 1(d) show the pattern of the parametric responses as a function of the antioxidant concentration. The parameters *τ* and *λ* show a linear behavior, while the rate parameters of *v*_*τ*_ and *v*_*m*_ display a decreasing asymptotic hyperbolic relation. Any of those parameters can be easily described via simple functions. Such a description produces detailed information, summarized in parameters that can be used to compare the AC of compounds, as an example.

Most of this information is already well-known [24, 30, 41]. However, authors [30, 32, 36] obtain the *τ* parameter, the most consistent one, by graphical analysis, and then a linear analysis is used to analyze the dose effect. Occasionally, (5) or similar are used [37] to extract the relevant information from the kinetic survival curve and then, the dose effect over the *τ* parameter is analyzed separately in linear terms. As it has being done in Figure 1, the analysis is performed in a two-step procedure. The statistical information of the parameters found in the first step is lost in the second one. Even if we compute the parametric estimations of the second step, it does not take into account the variable involved in the first step. Thus, among other problems, the two-step procedure lacks a proper statistical estimation.

When the kinetic OxHLIA survival responses were produced in the test-tube format, simple approaches seem to be acceptable. However, since the development of the microplate procedure, effortless time-dose data can be obtained and more complex or robust solutions are recommended. Next, general alternatives, including those to describe the antioxidant responses to build simultaneous solutions for most typical modifications of the kinetic erythrocyte survival curve by common tested effectors, are discussed.

### 3.3. Mathematical Model of Effectors Altering the Kinetic Description of the Survival Erythrocyte Population

The development of a theoretical model is greatly facilitated by the possibility of combining all experimental data into a single master curve that is able to account for the important variables simultaneously. Such a solutions allows us to control most factors that affect the system, helping to standardize the key variables for producing reproducible protocols and therefore, to obtain reproducible results. Despite the existence of very rigorous results regarding the kinetics and the factors affecting the kinetic curve of the survival erythrocyte population, simultaneous solutions are not always performed, and by applying them, we can provide key knowledge to understand partially the governing mechanisms.

#### 3.3.1. Possible Effector Variations on the Kinetic Description of the Survival Erythrocyte Population

We considered an effector as a variable or chemical entity that can cause a shape variation on the kinetic curve of the survival erythrocyte population, for example, the surfactant or NaCl concentration, typically applied in the osmotic fragility test; environmental variables such as pH and temperature; prooxidants such as AAPH; antioxidant agents. Unfortunately, due to the diversity and complexity of the perturbations of those effectors over the survival erythrocyte population curve, it is complex to postulate one empiric model that could be applied indiscriminately to produce all corresponding shape variations. The perturbations caused can be very different and affect one or various parameters of (5) (or any other reparametrized form). In general terms, four of the most common perturbations (linear, hyperbolic, sigmoidal, and bell modifications) over the kinetic curve of the erythrocyte survival population are summarized in Table 3 (part B).

Those pattern descriptions suggest different mechanisms of the cell lysis process. However, as in the previous section, because of the heterogeneity of the mechanistic reactions, it is difficult to characterize in mechanistic terms the hemolysis. Therefore, modeling with phenomenological approaches is the most appropriate solution to consider. When the previous phenomenological models are used we are not uncovering the mechanistic relations, but the hemolytic responses are a complex set of sequential and parallel reactions that represent, to an extent, one of the phenomenological curves.

In this regard, linear variations over the parameter *τ* have been found when describing the antioxidant inhibitory effects over the erythrocyte survival curve [24, 26, 30]. Hyperbolic ones have been found, when describing the effects of prooxidant agents, such as AAPH [36]. Sigmoidal effects also have been found, when describing the concentration of the surfactant or agents such as NaCl or the toxic palytoxin [28, 38, 40]. Bell profiles are normally found when analyzing the effects of variables such as temperature or pH [28].

The reader should note that the equations available to describe *H*, *S*, and *B* relations are diverse. We have selected the ones that we felt appropriate, but many different ones could be used.

#### 3.3.2. Simultaneous Solution to Describe the Effector Modifications over the Kinetic Curve of the Survival Erythrocyte Population: A Bivariate Approach

To introduce any of the previous phenomenological effector functions (*f*) affecting the survival erythrocyte (5), but without altering the kinetic parameters, such *f* should multiply any of its parameters (*θ*) as follows:

(6) $$\begin{array}{c} m_{\theta }=\theta_{C}\times \left[1+f\left(e\right)\right];\;\text{where}\;\left(\theta =K,\tau ,\alpha \right), \end{array}$$

in which *m*_*θ*_ is the modified final function for the parameters *θ* present in (5). Thus, when any parameter *θ* is perturbed by *f*(*e*), it becomes also a function of the variable *e*, but its original value without perturbation (or control, *θ*_*C*_) remains intact, and so does its statistical information. Therefore, a bivariate equation can be formulated, as a function of *t* and the modifier *e*, increasing significantly the descriptive capabilities of the model for real cases. In its more complete case, the proposal would be as follows:

(7) $$\begin{array}{c} P\left(t,e\right)=m_{K}\exp\left[-\ln2{\left(\frac{t}{m_{\tau }}\right)}^{m_{\alpha }}\right]. \end{array}$$

In consequence, when an entire set of kinetic profiles is simultaneously described by (7), the term *m*_*θ*_ typifies the specific variation, characterized by the mechanism caused due to the effector variable. Such a characterization allows using one (in the case of *L* relation) or various parameters (for *H*, *S*, or *B* relations) of *m*_*θ*_ and its statistical information for different purposes. This multivariable characterization is especially robust, minimizing the effects of random and systematic errors. As stated by many authors before [44, 51], optimally and efficient data analysis should involve simultaneous description of all curves, rather than fitting each one individually. The simultaneous curve-fitting reduces the number of parameters needed to analyze the response, which is a more informative approach and provides better estimations of parameters, and finally reduces their interval of confidence [31]. In addition, once all the modes of action are mathematically known, if the experimental curves obtained do not span the full range and some of them fail to provide information about one or more of the parameters of the equation, the bivariate application describes simply and accurately all the responses.

The reader should note that we have selected (5) as the principle model to perform the analysis of the effector agent. Thus, we have selected the form that makes explicitly the *τ* parameter and avoided the other possible reparameterization forms that provide other parameters of interests (*v*_*m*_, *v*_*τ*_, and *λ*). Such a preference, shared by many other authors in many different fields, should not be a restriction. Those other reparameterization forms could be used, but the essence of the simultaneous approach should be kept.

In the following, the specific OxHLIA survival responses produced by the action of antioxidant effectors are analyzed in detail first, in which a simple simultaneous time-dose model is presented. The model is verified using kinetic data effects of dose response of several antioxidants collected from the bibliography. Secondly, other typical modifications by other effectors are described in bivariate terms, extending the versatility and applicability of simultaneous approaches.

### 3.4. Verification of the Bivariate Procedure with Experimental Data from Bibliographic Results to Describe Antioxidant Agents as Effectors

**Figure 2:**
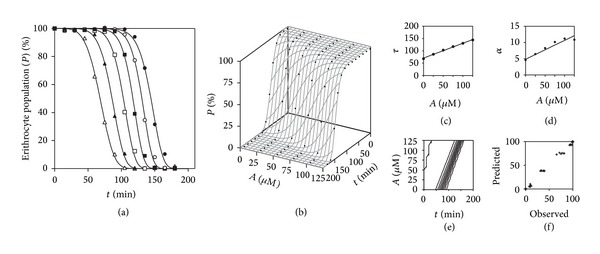
Trolox antioxidant case (Case A1, Table 1), presented in Figure 1 and analyzed simultaneously in terms of time and the dose by model (7). Statistical results in part A of Table 5 (Case A1).

An oxidation inhibitor can be characterized in detail by the variations caused in the parameters of (5), all of them with precise meanings, as well as theoretical and practical interest with respect to the oxidation kinetics. In open systems, the asymptotic parametric value (*K*) will always reach the maximum oxidizable value (in this case the 100% of the erythrocyte population). Thus, as a function of an A effector, the *K* parameter should not be modified by its action and should remain constant. Regarding the parameters *τ* and *α*, as suggested by the authors [24, 30], they should be modified by *L* relation. Therefore, by inserting relation ((iv) Table 3) modifying the parameters *τ* and *α* of (5), a bivariate model is obtained, as a simultaneous function of time and the modifier concentration.  Figure 2 shows the antioxidant (Table 1, Case A1) presented in Figure 1, analyzing simultaneously the time and the dose. The statistical results are displayed in part A of Table 5 (Case A1). From such determination, the parameter *l*_1_ (min/*μ*M of trolox), modifying the *τ* parameter of the kinetic curve of the survival erythrocyte population, provides the key relevant information for AC comparisons.

**Figure 3:**
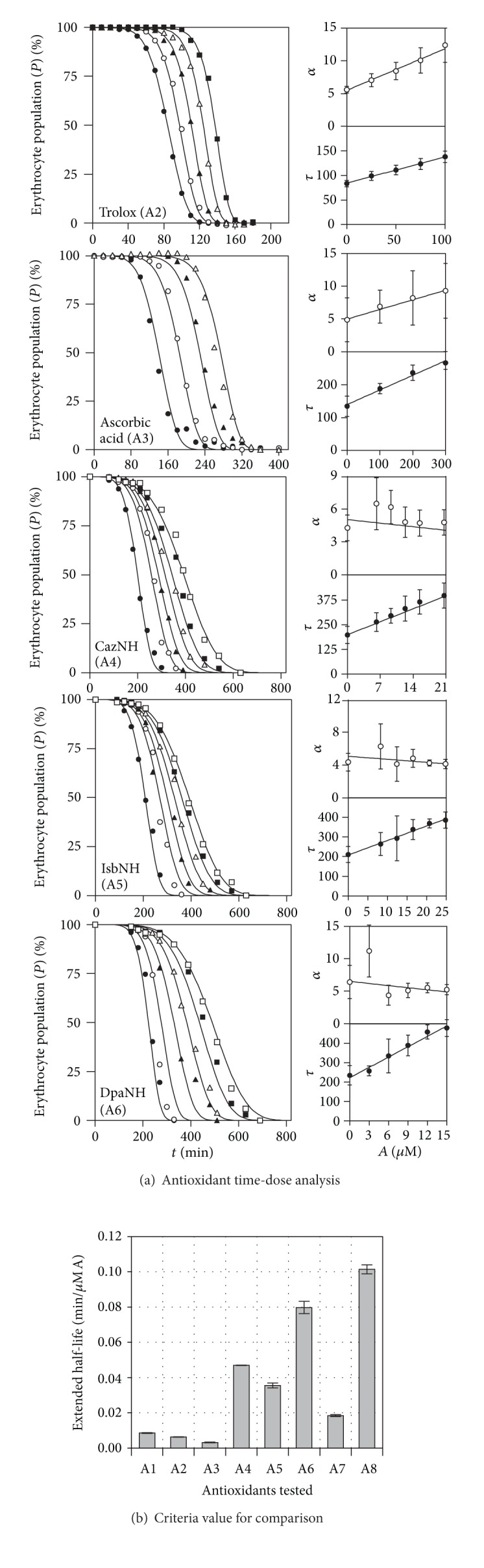
Graphical representation of the time-dose antioxidant responses collected from the bibliography (enumerated in Table 1) adjusted to (7) perturbed by (iv) (Table 3).

When we assume that the half-life (*τ*) is linearly modified by the presence of an antioxidant, such a behavior translates that each antioxidant molecule is able to capture the same amount during the time of the assay, independently of the type and the degree of polarity of the radicals generated. Also, it implies that in solution the antioxidants stability remains persistent during the time of the assay. This pattern has been found in other kinetic responses from different methodologies [41].  Figure 3 shows the analysis for other five cases collected from literature data (A2–6, Table 1). As in the previous case, the data could be modeled by inserting the linear relation ((iv) Table 3) modifying the parameters *τ* and *α* of (5). Parametric estimations and statistical information of the fittings are found in Table 5. Linear variations are successfully applied to the data published by different authors, and when we simultaneously allowed nonlinear relations, such as *H* or *S*, occasionally some improvements are obtained (data not showed). However, this saturated effect was weak and most of the attempts showed a nonsignificant solution of at least one parameter. The nonlinear pattern descriptions could uncover a saturated effect over the hemolytic reaction. This saturated response behavior has been already described in similar antioxidant responses by means of a hyperbolic effect over one or several parameters of the equation describing the kinetic oxidation, for example, the responses described in the LDL [52], *β*-carotene [53], and crocin [54] assay reactions to evaluate antioxidants. In any case, the apparently consistent linearity of the responses provides a simple solution to quantify the OxHLIA survival responses (Figure 3 and Table 5).

### 3.5. Extending the Analysis to Other Effectors

It is known that other effectors, such as metals, surfactants, and temperature or light intensity, are harmful to cell membranes, damaging its structure and eventually causing hemolysis. Such effectors, such as the antioxidant concentration, are dose-time dependent. Those effectors can be the interest in the AC determination since they can also destabilize AC. Understanding and describing its effect over the kinetic erythrocyte population curve are crucial in order to be able to quantify the changes that those effectors may produce.

More specifically, surfactants not only cause hemolysis, but also are commonly used to help hydrosoluble compounds to be solubilized in lipophilic environments or inversely. In this sense, in the OxHLIA analysis, researches could be interested to investigate how a hydrophilic A may be able to penetrate beyond the surface of the erythrocyte membrane, by adding different concentrations of a surfactant while studying the time-dose effect of an A. We have not found any bibliographic data available, but there are a plenty of studies that deal with the hemolytic effect of surfactants. Therefore, by being able to describe its effect in time- and effector-dependent form, we are providing the basis for postulating more complex multivariable models (time, antioxidant, and surfactant dose effect). Also in the case of surfactants, other agents or variables may be of interest to determine its effect to provide the base for more intriguing effects over the AC of compounds in the OxHLIA, such as the temperature or prooxidant agents.

Although the modifications produced by other variables (or effectors) over the kinetic erythrocyte population curve have been studied in detail and in many diverse reactions, this bibliographic data in many occasions is incomplete in the variable range studied or the effect is mixed with other variables. In addition, the data frequently is presented only in a 3D graph, which makes its data digitization nearly impossible. Thus, after an extensive search, only a small quantity of available examples could be used to test the general applicability of the general master model proposed.  Table 1 (part B) shows the examples selected. They involved different effectors of different hemolytic assays, affecting the kinetic survival erythrocyte population curve.

**Figure 4:**
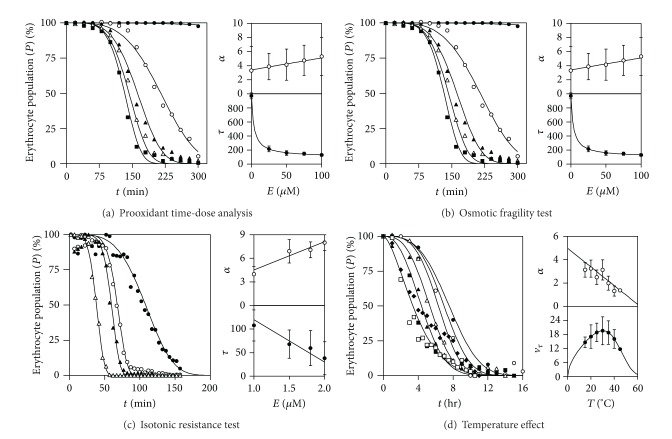
Graphical results of applying the kinetic model (5) or any other reparameterization form to the survival erythrocyte population curve affected by different effectors varying one or more parameters with one or various of the phenomenological functions described—equations ((iv), (v), (vi), and (vii); Table 3).

The results of applying kinetic model (5) or any of its other reparameterization forms to the survival erythrocyte population curve affected by different effectors varying one or more parameters with one or various of the phenomenological functions described—Equations ((iv), (v), (vi), and (vii) Table 3)—are presented in Figure 4 independently of the mechanistic interpretation that can be inferred by analyzing the specific behavior of the characterizing parameters of (5) and modified by the actions of effectors. The time and effector dependent functions are used, and this produces consistent and meaningful criteria to characterize and quantify the survival erythrocyte population minimizing the effects of the error produced by the experimental conditions.

In this regard, when studying the activity of prooxidant agent AAPH [36] (E1, Table 1) over the kinetic erythrocyte population curve (Figure 4(a)), *H* decreasing behavior affecting the parameter *τ* and *L* increasing relation for the parameter *α* are found. Those phenomenological effector responses over the erythrocyte population curve traduce a saturated effect produced by the AAPH agent.  Figure 4(b) shows a typical response in the osmotic fragility test caused by the surfactant DATB [38] (E2, Table 1). The parametric modifications over the parameters are inversely related to those found for the previous case (*H* decreasing relation for *α* and *L* increasing one for *τ*). When observing the isotonic resistance test responses [39] (E3, Table 1), using increasing concentrations of ammonium chloride (Figure 4(c)), *L* modifier relations are found for the parameters *τ* and *α*. Finally, using the effect of temperature over kinetic erythrocyte population curve [28], Figure 4(d) shows the case in which a bell type curve is found useful to characterize the effector pattern. For example, we have used the reparametrized form (9), making explicitly the parameter that accounts for the rate of the process when the 50% of the population are lysed (*v*_*τ*_, % erythrocyte lysis/hr). The value *v*_*τ*_ is modified by B type function and the *α* by L decreasing one. For all the assayed effectors, statistically significant descriptions, with very accurate predictions, were provided.

## 4. Conclusions

We have established that the kinetic model (5) (or any of its other reparameterization forms) describes adequately the variation of the degree of hemolysis as a function of the time. Afterwards a simple bivariate analysis taking into account the time and the antioxidant dose was successfully modeled by means of a simultaneous analysis. Overall, in this paper, a quantification method was developed for the OxHLIA and verified by analyzing the results of other authors that had investigated the capacity of several antioxidants. This analysis reveals the high efficiency of the proposed approach. The model parameters obtained were used to compare the capacity, identify trends, and analyze the dose-equivalent system response, providing more complete information about antioxidant behavior. Therefore, a more efficient way to determine total antioxidant capacities than the simple approach to study the dose-response in a two steps procedure is presented.

In addition, bivariate analysis taking into account the time and other effectors was successfully modeled in similar principles more than those used for antioxidant responses. Using a bivariate model seems to be a disadvantage regarding other apparent simpler routines. In agreement with the opinion of Niki et al. [24], we believe that bivariate approaches of the type proposed here could improve any bioassay in which the inhibitory or stimulatory action of an effector is superposed onto the variation with time of the target system, or the particular time-course of the response is a relevant aspect of that action. Most bioassays that are based on hemolytic processes could be examples on this matter, as it has been demonstrated and discussed above in detail.

## Figures and Tables

**Table 1:** Description of the references used to collect data to be analyzed in this study, the figures, variable or agent involved, and some relevant conditions of the assays.

Case	Reference	Figure	Effector	Conditions	Method
A: Antioxidant effector analysis					
A1	[26]	Figure 20	Trolox (0-(25)-125 *μ*M).	0.7% (v/v) in PBS (pH 7.4), 37°C, 40 mM AAPH.	OxHLIA^a^
A2	[30]	Figure 3	Trolox (0-(25)-100 *μ*M).	0.7% (v/v) in PBS (pH 7.4), 37°C, 40 mM AAPH.	OxHLIA^b^
A3	[36]	Figure 5	Ascorbic Acid (0-(100)-300 *μ*M).	0.7% (v/v) in PBS (pH 7.4), 37°C, 50 mM AAPH.	OxHLIA^a^
A4	[37]	Figure 1(a)	CazNH (0, 6.17, 9.26, 12.3, 15.4, 20.6 *μ*M)	10 mM PBS (pH 7.4), 37°C, 40 mM AAPH.	OxHLIA^a^
A5	[37]	Figure 1(b)	IsbNH (0, 8.26, 12.4, 16.5, 20.7, 24.8 *μ*M)	10 mM PBS (pH 7.4), 37°C, 40 mM AAPH.	OxHLIA^a^
A6	[37]	Figure 1(c)	DpaNH (0, 3, 6, 9, 12, 15 *μ*M)	10 mM PBS (pH 7.4), 37°C, 40 mM AAPH.	OxHLIA^a^
A7	[37]	Figure 1(d)	PtzNH (0, 20.3, 30.5, 37.3, 50.8, 61.0 *μ*M)	10 mM PBS (pH 7.4), 37°C, 40 mM AAPH.	OxHLIA^a^
A8	[37]	Figure 1(e)	PozNH (0, 9.27, 10.8, 12.4, 13.9, 15.5 *μ*M)	10 mM PBS (pH 7.4), 37°C, 40 mM AAPH.	OxHLIA^a^

B: Another effector analysis					
E1	[36]	Figure 3	AAPH (0-(20)-100 *μ*M).	0.7% (v/v) in PBS (pH 7.4), 37°C.	OxHLIA^a^
E2	[38]	Figure 1	DTAB surfactant (2.25, 4.5, 5.625, 6.75, 11.25%).	10 mM PBS (pH 7.4), 37°C.	Osmotic fragility test^a^
E3	[39]	Figure 7	NaHCO_3_ (1, 1.5, 1.8, 2 mM).	pH 7.2, 20°C.	Isotonic hemolytic test^a^
E4	[28]	Figure 4	Temperature (15, 20, 25, 30, 35, 40, 45°C).	10 mM PBS (pH 7.4), palytoxin (0.25 pg/*μ*L).	Palytoxin hemolytic activity^c^

^a^Test-tube format.

^b^Microplate format.

^c^Half-way between test-tube and microplate format.

**Table 2:** Short review of different mathematical methodologies from related fields of study, such as the hemolytic bioassays, antioxidant, and dose-response theory.

Reference	Type∗	Use∗∗	Description
From related hemolytic analytical techniques			
[39]	M	OMT	Sophisticated mechanistic model to evaluate the erythrocyte lysis analyzed with a scanning flow cytometer in isotonic solution, obtaining several parameters (volume, surface area, hemoglobin concentration, elasticity. and critical tension of membrane, etc.) that allow us to evaluate the lysis.
[40]	E	OMT	A mathematical model based on the Gaussian distribution function to measure the degree of osmotic fragility to test the degree of resistance of red blood cells to hemolysis was developed. It provides parameters that define the midpoint, the dispersion, and maximum hemolysis, respectively.
[38]	E	OMT	Demonstrating the suitability of the Weibull survival distribution to study the surfactant-induced erythrocyte hemolysis (osmotic fragility test) connecting its parameters to blood properties.
[28]	E	PHA	Developing a toxicological dynamic model, applying in equivalent form the Weibull and Logistic equation, to describe the hemolysis of erythrocytes by palytoxin and its inhibition by ouabain, allowing us to detect this potentially nonprotein marine toxin.

From the antioxidant field			
[41]	E	DAA	A bivariate model was proposed. It allows us to obtain the simultaneous solution of a series of oxidation kinetics of a dose-response of antioxidants. Its application is simple, provides parametric estimates which characterize oxidative process, and facilitates rigorous comparisons.
[42]	E	DAA	A kinetic approach to evaluate the efficiency of antioxidants in scavenging the radical generated in the *β*-carotene, DPPH, and the superoxide anion radical methods. The authors highlighted the need of approaches to estimate the rate of the antioxidant reactions.
[43]	E	DAA	A general mathematical model for lipid oxidation in food systems based on the logistic equation. A simple method was described for the evaluation of the model parameters. Variations of these numerical values were also associated with varying pretreatment and storage conditions.

From the dose-response theory			
[44]	E	DDRA	A general bivariate method to describe the time-dose-response curves for physiological and pharmacological studies. The method permits rigorous statistical analysis, provides a basis for pooling of information from separate experiments, and determines characteristics shared by curves.
[45]	E	DDRA	A review to describe the importance of the time dimension on dose-responses for toxic chemicals. In many situations, the effect of a toxic chemical on a biological system depends on both the intensity and the duration of exposure.
[46]	E	DDRA	The suitability of several common descriptive models for the study of dose-response relationships is discussed, and changes are introduced that improve their suitability, generalize their application, and lead to their possible application for multivariable analysis.
[47]	E	DDRA	A review of various properties of the Hill equation which is widely used in many pharmacokinetic-pharmacodynamic models to describe nonlinear drug dose-response relationships. The main mechanistic aspect and multivariate potential applications are also discussed.

*Model type: mechanistic (M); empirical (E).

**Use: Osmotic fragility test (OFT); palytoxin hemolytic activity (PHA); different antioxidant assays (DAA); different dose-response approaches (DDRA).

**Table 3:** Part A shows the additional parameters of interest (*v*_*m*_, *v*_*τ*_, and *λ*) and reparameterization equations deduced from algebraic modifications of (5) to make such values explicit and therefore to compute their confidence intervals. Part B shows four of the most common effector perturbations (linear, hyperbolic, sigmoidal, and bell modifications) on the kinetic description of the survival erythrocyte population.

A: Additional parameters of interest		
Parameter calculation	Reparameterization form of (5)	Eq. number
$v_{m}=\frac{K\alpha \ln2}{\tau }G^{G}e^{-G}$	$P=K\exp\left[-{\left(\ln2\right)}^{1-\alpha }{\left(\frac{e^{G}v_{m}t}{K\alpha G^{G}}\right)}^{\alpha }\right]$	(i)
$v_{\tau }=\frac{K\alpha \ln2}{2\tau }$	$P=K\exp\left[-{\left(\ln2\right)}^{1-\alpha }{\left(\frac{2v_{\tau }t}{K\alpha }\right)}^{\alpha }\right]$	(ii)
$\lambda =\frac{\tau }{\sqrt[{\alpha }]{\ln2}}\left[G^{1/\alpha }+\frac{e^{-G}-1}{\alpha G^{G}e^{-G}}\right]$	$P=K\exp\left[-{\left(\left(G^{1/\alpha }+\frac{e^{-G}-1}{\alpha G^{G}e^{-G}}\right)\frac{t}{\lambda }\right)}^{\alpha }\right]$	(iii)

B: effector variations on the survival population		
Effector relation	Model chosen to modify parameters of (5)	Eq. no.

Linear (*L*)	*L*(*e*) = *e* · *l*_1_ + *l*_2_	(iv)
Hyperbolic (*H*)	*H*(*e*) = *h*_1_[1 − exp⁡(*h*_2_ · *e*)]	(v)
Sigmoidal (*S*)	*S*(*e*) = *s*_1_exp⁡[−ln⁡2(*e*/*s*_2_)^*s*_3_^]	(vi)
Bell (*B*)	$B\left(e\right)=b_{1}\left\{\frac{b_{3}}{b_{2}}\left[1-{\left(\frac{e}{b_{4}}\right)}^{b_{2}}+\ln\left(\frac{e}{b_{4}}\right)b_{2}\right]\right\}$	(vii)

Part A: maximum rate of hemolysis (*v*_*m*_); the rate at the value (*v*_*τ*_); the lag-phase (*λ*); and *G* = (*α* − 1)/*α*.

Part B: *l*_1_ is the slope (*t*/*e* units); *l*_2_ is the intercept (*t* units); *h*_1_ is the asymptotic value of the hyperbolic relation (parameter modified units); *h*_2_ is 1/*e* units;*s*_1_ is the asymptotic value (parameter modified units) of the nonlinear relation, *s*_2_ is the IC_50_ value (*e* units); *s*_3_ is a shape parameter related to the maximum slope of the response; *b*_1_ is the maximum value (parameter modified units), *b*_2_ is related to the distance between the tails of the function (*e* units), *b*_3_ is a value related to the asymmetry of the bell profile, and *b*_4_ is the effector value at which *b*_1_ takes place (*e* units).

**Table 4:** Parametric estimations and statistic information of the kinetic series of the survival erythrocyte population inhibited by the antioxidant trolox and fitted to the kinetic model (5).

Effector	Kinetic parameters			Statistics	Reparameterization		
Trolox (*μ*M)	*K* (% Ert)	*τ* (min)	*α*	*R*_adj_^2^	*v*_*τ*_ (% Ert/min)	*v*_max⁡_ (% Ert/min)	*λ* (min)
0	100 ± 1.15	68.12 ± 10.03	4.59 ± 2.48	0.9992	2.34 ± 1.01	2.35 ± 0.88	39.92 ± 12.12
25	100 ± 1.15	86.05 ± 10.63	6.44 ± 5.17	0.9993	2.59 ± 0.98	2.63 ± 0.47	59.87 ± 17.22
50	100 ± 1.15	102.96 ± 10.74	8.26 ± 7.73	0.9984	2.78 ± 0.75	2.85 ± 0.65	78.16 ± 21.34
75	100 ± 1.15	117.26 ± 10.53	10.14 ± 6.89	0.9988	3.00 ± 0.68	3.09 ± 0.32	94.04 ± 18.75
100	100 ± 1.15	130.76 ± 10.47	11.20 ± 6.92	0.9987	2.97 ± 0.59	3.06 ± 0.89	107.22 ± 21.24
125	100 ± 1.15	144.26 ±10.60	10.86 ± 8.30	0.9971	2.61 ± 0.85	2.69 ± 0.97	117.51 ± 31.25

**Table 5:** Numeric results corresponding to the time-dose antioxidant responses collected from the bibliography (enumerated in Table 1) adjusted to (7) perturbed by (iv) in Table 3.

		Parametric estimates					Statistics
Effector		Kinetic parameters			Effector modifying coefficients		*R*_adj_^2^
		*K*	*τ*	*α*	*τ* modifier (*l*_1_)	*α* modifier (*l*_1_)	
A1	Trolox	100.0 ± 0.5	70.5 ± 0.9	4.83 ± 6.9	0.0086 ± 2.2	0.0121 ± 16.5	0.9973
A2	Trolox	100.0 ± 0.3	84.2 ± 0.4	5.50 ± 3.6	0.0063 ± 1.4	0.0115 ± 9.7	0.9993
A3	Ascorbic Acid	100.0 ± 1.1	139.8 ± 1.8	4.97 ± 12.4	0.0032 ± 4.6	0.0030 ± 37.2	0.9937
A4	CazNH	100.0 ± 1.1	199.7 ± 1.3	5.03 ± 12.2	0.0470 ± 0.3	−0.0092 ± 11.9	0.9877
A5	IsbNH	100.0 ± 1.0	208.1 ± 1.4	5.07 ± 7.5	0.0356 ± 3.9	−0.0073 ± 46.8	0.9884
A6	DpaNH	100.0 ± 1.7	222.6 ± 1.8	6.48 ± 11.5	0.0798 ± 4.4	−0.0169 ± 47.8	0.9713
A7	PtzNH	100.0 ± 0.8	209.0 ± 1.2	6.65 ± 7.9	0.0184 ± 2.9	−0.0031 ± 47.4	0.9892
A8	PozNH	100.0 ± 0.9	186.5 ± 1.3	9.09 ± 11.3	0.1014 ± 2.6	−0.0295 ± 20.6	0.9890

Confidence intervals for *α* = 0.05. *R*_adj_^2^: adjusted determination coefficient.
